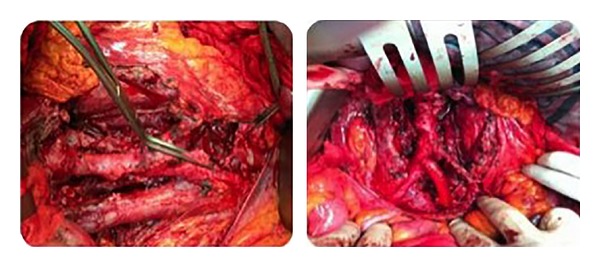# Growing Teratoma Syndrome

**DOI:** 10.1155/2014/139425

**Published:** 2014-08-17

**Authors:** Anna Scavuzzo, Zael Arturo Santana Ríos, Nancy Reynoso Noverón, Miguel Angel Jimenez Ríos

**Affiliations:** Department of Urology, Instituto Nacional de Cancerología (INCan), Avenida San Fernando 22, Colonia Seccion XVI, Tlalpan, 14080 Mexico City, DF, Mexico

## Abstract

Growing teratoma syndrome (GTS) is a rare clinical entity, which presents with enlarging teratomas masses of the retroperitoneum or other locations, occurring during or after systemic chemotherapy for the treatment of nonseminomatous germ cell of the testis (NSGCT), with normalised tumour markers. Awareness of this syndrome is necessary in order to prevent unnecessary chemotherapy and allow optimal management. Prognosis is excellent after the excision of these tumors, but surgery has to be as complete as possible. Surgical resection of bulky GTS lesions is technically challenging; intraoperative complications may occur; that is, why the treatment must not be delayed. Our experience in the surgical management of these lesions is reviewed in the following work.

## 1. Introduction

Growing teratoma syndrome (GTS) is a rare condition related to both testicular and ovarian carcinoma. The incidence of GTS after testicular NSGCT is 1.9–7.6%, while in the setting of ovarian germ cell neoplasia is unknown [1]. It is characterized by an increase in metastatic mass caused by mature teratoma in patients with no viable germ cell tumor during or after chemotherapy. According to Logothetis criteria [2], the definition of GTS includes (1) normalization of serum tumor markers, alpha fetoprotein, and human chorionic gonadotropin; (2) enlarging or new masses despite appropriate chemotherapy for nonseminomatous germ cell tumors; and (3) the exclusive presence of mature teratoma in the resected specimen. Surgical treatment and prognosis are highly dependent on the timing of diagnosis [3].

## 2. Case Presentation

### 2.1. Case 1

A 23-year-old man underwent right orchiectomy and four cycles of bleomycin, etoposide, and platinum chemotherapy at another hospital for T2 mixed NSGCT (50% teratoma, 30% choriocarcinoma, and 20% embryonal carcinoma). The initial clinical stage of disease was IIC. He was classified, according to International Germ Cell Cancer Collaborative Group (IGCCCG) classification, as the intermediate prognostic group.

**Figure 1:**
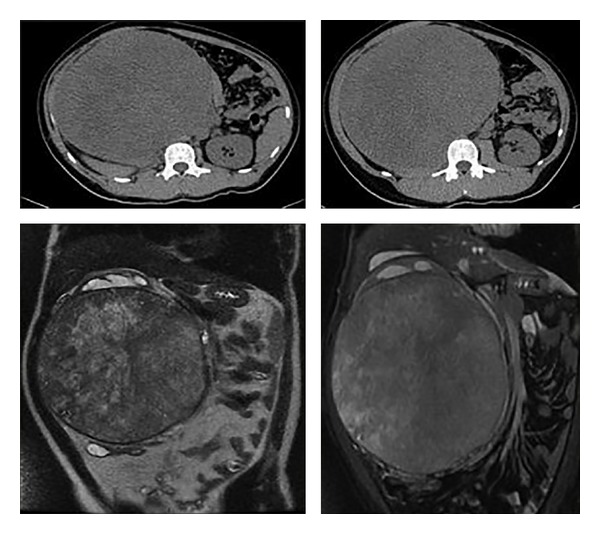


**Figure 2:**
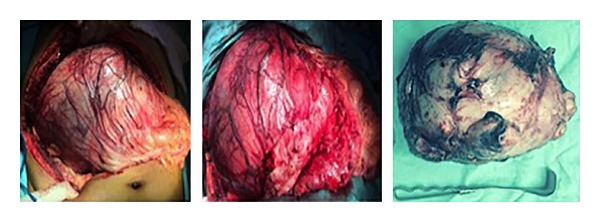


After detection of unresectable tumor during postchemotherapy RPLND, 12 months after orchiectomy, the patient was referred to our hospital. A computerized tomography (CT) scan revealed a giant mass with a diameter of 21 cm which displaced the inferior vena cava, right kidney, and left psoas muscle. MRI examination was performed in order to characterize the mass better and its relationship to adjacent structures. MRI images showed no infiltration IVC (Figure 1). His *α*-fetoprotein was 2.1 ng/mL, lactate dehydrogenase was 122 IU/L, and beta subunit of human chorionic gonadotropin was 0 mIU/mL. The tumor was completely resected (Figure 2). Surgery was performed through transverse abdominal incision. Right nephrectomy was deemed necessary because no dissection plane remained between the residual tumor and the kidney. Operative time was 2,5 hours with no required transfusion. A fluid diet was started on the first postoperative day. Postoperative period was uneventful. He remained hospitalized for 3 days. Histologic find was teratoma mature. After a follow-up of 10 months, the patient is alive without recurrence.

### 2.2. Case 2

**Figure 3:**
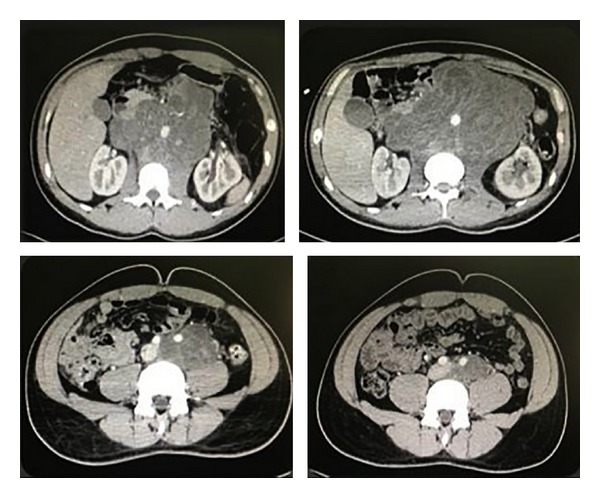


A 28-year-old man with a history of left orchiectomy for mixed NSGCT (80% teratoma and 20% embryonal carcinoma) without adjuvant therapy, six months later, started having gastrointestinal symptoms, weight loss, and lower back pain as well as abdominal mass. Workup revealed that *α*-fetoprotein, lactate dehydrogenase, and beta subunit of human chorionic gonadotropin levels were 2372 ng/mL, 216 IU/L, and 395 mIU/mL. The chest radiography shows metastatic lesions. Abdominal CT evidenced giant retroperitoneal tumor from the renal vessels to the left iliac vessels. The clinical stage was IIIA for cervical lymph nodes. He received three cycles of bleomycin, etoposide, and platinum chemotherapy until normalization of serum tumor markers. CT scan showed an increase in tumor size (30 cm) after five months from chemotherapy (Figure 3) and chest metastasis disappearance. Tumor was resected through transverse incision. Iliac vessels were resected in block and continuity was restored by Dacron graft without complications (Figure 4). Closed drainage system was left in the retroperitoneal space at the end of the procedure. Estimated blood loss was greater than 2,500 mL. Operative time was 6 hours. The patient required a transfusion of 2 units of packed red blood cells during surgery. He was admitted to the intensive care unit after surgery and was dismissed from the intensive care unit on postoperative day 1. A fluid diet was started on the third postoperative day and solid diet on the fifth. He was discharged home postoperatively after six days in good general condition. Abdominal drains were removed when daily output was below 100 mL. Histologic find was teratoma mature. After follow-up of 8 months, he had no recurrence.

## 3. Discussion

The etiology of GTS is unknown but there are two must-quoted hypotheses: chemotherapy cures immature malignant cells but remains untreated and grows the mature benign teratomatous elements; chemotherapy alters the cell kinetics toward transformation from a totipotent malignant germ cell toward a benign mature teratoma [1].

While the growing teratomas are considered benign, they have rapid expansion with median linear growth of 0,5–0,7 cm/month and volume increase of 9,2–12,9 cm^3^/month, though the growth patterns are variable [3, 4]. Their behaviour is unpredictable for aggressive local spread and potential malignant degeneration [2, 3, 5].

This syndrome has been reported in the retroperitoneum (the most common site), lung, cervical lymph nodes, mediastinum, supraclavicular lymph nodes, inguinal lymph nodes, forearm, mesentery, liver, and pineal gland [6, 7].

According to André et al. [8], there are elements that predict the development of GST: the presence of teratoma mature in the primary NSGCT, no decrease in the size of tumor during chemotherapy; the presence of teratoma in postchemotherapy residual masses. So, close follow-up by radiological imaging, possibly after 2 cycles of chemotherapy, for early recognition of GST is necessary in patients with factors risk [3, 8]. Keep in mind that when surgery is delayed, prevalent complications are caused by local compression, including obstructive renal failure or bowel, duodenal, bile duct, or large vessel obstruction [3]. Expeditious surgery is important, as with time, so it can develop unresectable disease.

Surgical resection is currently the gold standard treatment for GST, since teratomas are resistant to chemotherapy and radiotherapy [1].

Two described cases are bulky teratomas (defined as >10 cm) [9] with high volume that represent a technical challenge for possible intraoperative complications. In a series reported the volume of the tumor is associated with increased difficulty in dissecting the tumor mass from major vascular structures [9]; the risk of resection of vena cava and nephrectomy rate were 7,1 and 31,3%, respectively [10]. The involvement of vascular structure or other organs is not considered as contraindication for the surgery [8]. Still our cases suggest that it is possible to carry out the surgery procedure also in front of large tumors, taking into account the possibility of eventual resection and reconstruction of adjacent vascular structures. So we found that transverse abdominal incision is safe and an appropriate broader access rather a midline laparotomy. Aggressive surgery with resection of major abdominal vascular and visceral structure is necessary for giant tumors to obtain complete excision of retroperitoneal mass.

Various series supported that surgical treatment is curative and the local recurrence is lower when the tumor is completely removed [3, 8, 9]. Local recurrence may be attributable to inadequate and incomplete resection [3].

Data reported into two large studies, by Spiess et al., the 5-year overall survival were 89-90% in patients who underwent complete resection [3, 8].

## 4. Conclusion

Patients with advanced germ cell tumors should receive coordinated care by treating urologist and oncologist to recognize promptly GST and to achieve good outcomes. Complete surgical treatment is recommended, even if it is technically challenging, to avoid mechanical complication and malignant transformations. In cases of bulky teratomas, surgery should be performed at a highly specialized center with a skilled surgeon.

## Figures and Tables

**Figure 4:**